# Inhibition of LIPG phospholipase activity suppresses tumor formation of human basal-like triple-negative breast cancer

**DOI:** 10.1038/s41598-020-65400-7

**Published:** 2020-06-02

**Authors:** Pang-Kuo Lo, Yuan Yao, Qun Zhou

**Affiliations:** VA Maryland Health Care System, Department of Biochemistry and Molecular Biology, Greenebaum Cancer Center, University of Maryland School of Medicine, Baltimore, MD 21201 USA

**Keywords:** Cancer, Molecular medicine, Oncology

## Abstract

The endothelial lipase LIPG possesses serine phospholipase activity and is involved in lipoprotein metabolism. Our previous studies have revealed that LIPG overexpression is required for tumor formation and metastasis of human basal-like triple-negative breast cancer (TNBC). We also demonstrated that LIPG differentially regulates TNBC malignancy through its enzymatic and non-enzymatic functions. The present studies were aimed at determining how XEN445, a specific inhibitor targeting LIPG phospholipase activity, impacts on TNBC tumor formation and malignant features. We established a cell-based LIPG enzymatic assay system to measure the inhibitory effect of XEN445 on LIPG phospholipase activity and determine its IC50. We found that XEN445 preferentially inhibited the proliferation of LIPG-expressing TNBC cells but not LIPG-negative luminal breast cancer cells. XEN445 inhibited the self-renewal of cancer stem cells (CSCs) *in vitro* and TNBC tumor formation *in vivo*. However, XEN445 had no inhibitory effect on the invasiveness and CSC stemness of TNBC cells. Our studies suggest that targeting both LIPG enzymatic and non-enzymatic functions is an important strategy for the treatment of TNBC.

## Introduction

Lipases control lipid metabolism by regulating the hydrolysis of neutral lipids to free fatty acids (FFAs) and glycerol. FFAs generated from lipolysis are imported into the cell for energy usage and storage, where lipases are critically involved in regulating energy homeostasis. This lipase-mediated lipolysis is critical for the breakdown of extracellular lipoproteins, triglycerides, and phospholipids. One of lipases is LIPG (also called endothelial lipase), which is encoded by the gene *LIPG*. LIPG, first identified in 1999^1,2^, is a member of the triglyceride lipase family and shares significant protein sequence homology (about 40% identity) with other triglyceride lipases^1,2^. The LIPG protein sequence contains motifs (GXSXG, the heparin-binding motif, and the catalytic triad S169, D193, H274), which are critical for lipase activity^1,2^. LIPG manifests predominant serine phospholipase activity when compared with other triglyceride lipases (e.g., lipoprotein lipase (LPL) and hepatic lipase (HL)) that mainly display triglyceride lipase activity^3,4^. LIPG has shown to be responsible for the metabolism of several lipoproteins *in vitro* and *in vivo*, in particular high-density lipoprotein particles (HDL cholesterol)^3,4^. Adenovirus-mediated overexpression of LIPG in LDL receptor-deficient mice resulted in the reduction of plasma VLDL and LDL cholesterol levels by about 50%. However, it almost depleted plasma HDL cholesterol levels, indicating that LIPG plays a critical role in HDL catabolism^1^. HDL is known as “protective” cholesterol because having high HDL levels can reduce the risk of heart disease and stroke. Since LIPG can hydrolyze HDL phospholipids, the overabundance of LIPG leads to the lower circulating levels of HDL, which promotes heart disease. As blocking LIPG activity leads to an increase in total plasma HDL levels, inhibition of LIPG activity has emerged as a potential therapeutic strategy for the treatment of dyslipidemia-related cardiovascular disease. Therefore, LIPG inhibitors have shown therapeutic potential for cardiovascular disease. Several LIPG inhibitors have been identified, including sulfonylfuran urea 1^5^, phenylboronic acid derivatives^6^, thiocarbamate^7^ and XEN445^8^. Among these discovered inhibitors, XEN445 is a potent, selective LIPG inhibitor and has shown to raise the plasma HDL-cholesterol concentration in mice^8^.

Breast cancer, a heterogeneous disease, has been categorized into several molecular subtypes, including basal-like, HER2-positive, and luminal breast cancers^9,10^. Triple-negative breast cancer (TNBC)^11^, characterized by the triple negativity of estrogen receptor (ER), progesterone receptor (PR) and HER2, is highly aggressive and shows rapid tumor growth with high incidence of metastasis, and the poor clinical outcome^10,12,13^. Basal-like TNBC is highly related to an epithelial-mesenchymal transition (EMT) phenotype^14^, which plays important roles in tumor metastasis by stimulating the formation of CD44+ CD24- cancer stem cells (CSCs), enhancing tumor cell invasion, motility and chemoresistance^15^.

**Figure 1:**
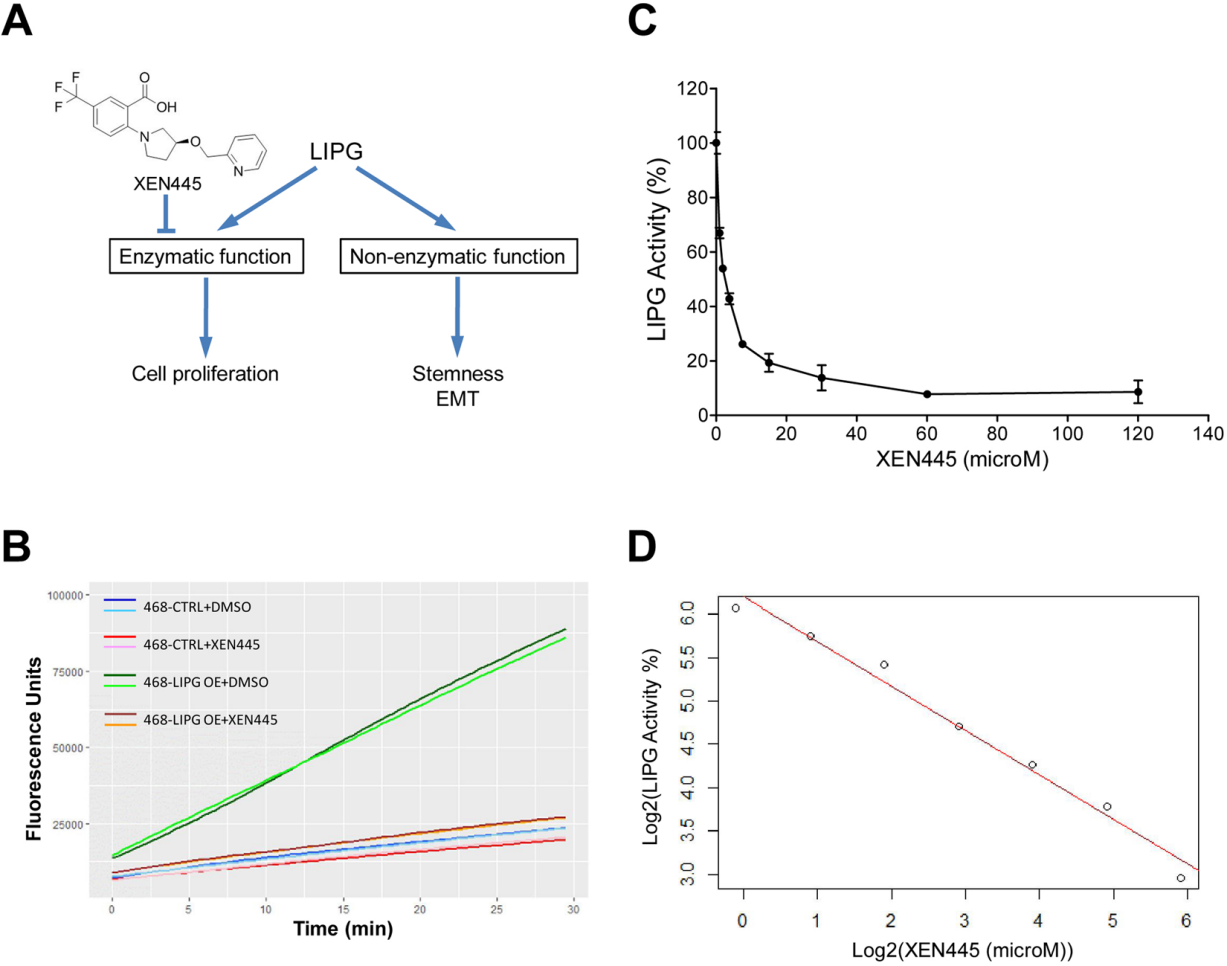
Analysis of the inhibitory effect of XEN445 on the phospholipase activity of LIPG in a cell-based assay. **(A)** A diagram shows the functional role of LIPG in breast cancer. LIPG possesses both enzymatic and non-enzymatic functions to specifically regulate different aspects of breast cancer malignancies. XEN445 is a pharmacological inhibitor that specifically targets the enzymatic function of LIPG. **(B)** Kinetically studying LIPG phospholipase activity of LIPG-overexpressing MDA-MB-468 cells with or without XEN445 treatment. Parental and LIPG-overexpressing MDA-MB-468 cells were analyzed in LIPG enzymatic assays. Either vehicle (DMSO) or XEN445 (200 μM) was included in assays. Kinetic studies were performed for 30 minutes with 1 cycle/min. Duplicate experiments were performed and their kinetic data were plotted separately for showing experimental accuracy and reproducibility. Two biological replicates for each assessed sample include 468-CTRL + DMSO (indicated by dark blue and sky blue), 468-CTRL + XEN445 (red and pink), 468-LIPG OE + DMSO (dark green and light green) and 468-LIPG OE + XEN445 (dark brown and orange). **(C)** Dose-dependent analysis of the inhibitory effect of XEN445 on LIPG phospholipase activity. The phospholipase activity of LIPG was measured under a series of 2-fold diluted XEN445 doses as indicated in the figure. The end-point enzymatic activity of LIPG was plotted against the XEN445 dose. The relative LIPG enzymatic activity expressed as a percentage was calculated from the measured enzymatic fluorescence data as described in “Materials and Methods”. Triplicate experiments were performed. Error bars shown in the plot are standard deviation (SD). **(D)** Linear regression analysis of log2-transformed LIPG enzymatic activity against log2-transformed XEN445 doses. Data shown in **(C)** were log2-transformed and their linear regression relationship was analyzed. The fitted linear regression line was plotted based on the linear modeled equation log2(LIPG activity) = −0.514 X log2(XEN445 dose) + 6.203 (multiple R-squared: 0.9825, adjusted R-squared: 0.979). The predicted IC50 is 2.172 μM.

Our recent studies have shown that LIPG is preferentially overexpressed in basal-like TNBCs when compared with healthy breast tissue and other breast cancer subtypes^16^. We have demonstrated that LIPG activated the oncogenic DTX3L-ISG15 signaling pathway leading to metastasis of basal-like TNBC^16^. Moreover, we have shown that LIPG possesses a lipase-dependent function to support TNBC cell proliferation and a lipase-independent function to promote invasiveness, stemness and basal/EMT features of TNBC (Fig. 1A)^16^.

Here, we reported that XEN445 selectively inhibited the enzymatic function of LIPG. In addition, we uncovered that XEN445 could impact the proliferation, invasiveness, stemness, and basal/EMT features of TNBC. These findings established the foundation for future investigation of LIPG as a therapeutic target for TNBC.

## Results

### Analysis of the inhibitory effect of XEN445 on the phospholipase activity of LIPG

XEN445 has shown to inhibit the phospholipase activity of LIPG (Fig. 1A)^8^. To validate the inhibitory effect of XEN445 on the enzymatic activity of LIPG at a cellular level, we performed LIPG enzymatic assays using parental and LIPG-overexpressing MDA-MB-468 cell lines^16^. Figure 1B shows that LIPG-overexpressing MDA-MB-468 cells (468-LIPG OE) manifested significantly higher phospholipase A1 (PLA1) activity than parental cells (468-CTRL) (approximately 5-fold). Treatment with XEN445 (200 μM) led to the approximate 80% reduction of PLA1 activity in 468-LIPG OE cells compared to the estimated 25% reduction of PLA1 activity in 468-CTRL cells (Fig. 1B). Given that 200 μM XEN445 nearly inhibited the total overexpressed LIPG activity and one-fourth cellular endogenous PLA1 activity, these results suggest that XEN445 is a specific LIPG inhibitor and endogenous LIPG in MDA-MB-468 cells contributes to approximate 25% of total PLA1’s activity. These findings indicate that the 468-LIPG OE cell line is a suitable cell model for the analysis of the XEN445 effect on LIPG enzyme activity.

We performed LIPG enzymatic assays to determine the IC50 of XEN445. Figure 1C shows the relationship between LIPG activity and XEN445 doses was not linear. Data shown in Fig. 1C were log2-transformed, and a linear regression relationship was analyzed. We found that log2 values for both variables (LIPG activity and XEN445 dose) showed a linear relationship with intercept (6.203), slope (−0.514) and adjusted R-squared (0.979) values (Fig. 1D). Based on the linear regression equation, the predicted IC50 of XEN445 is 2.172 μM, which is approximately 9-fold higher than the reported IC50 (0.237 μM) of XEN445^8^. This difference may be due to a different approach used in the assay system.

### XEN445 preferentially inhibits the proliferation of LIPG-expressing TNBC cells

**Figure 2:**
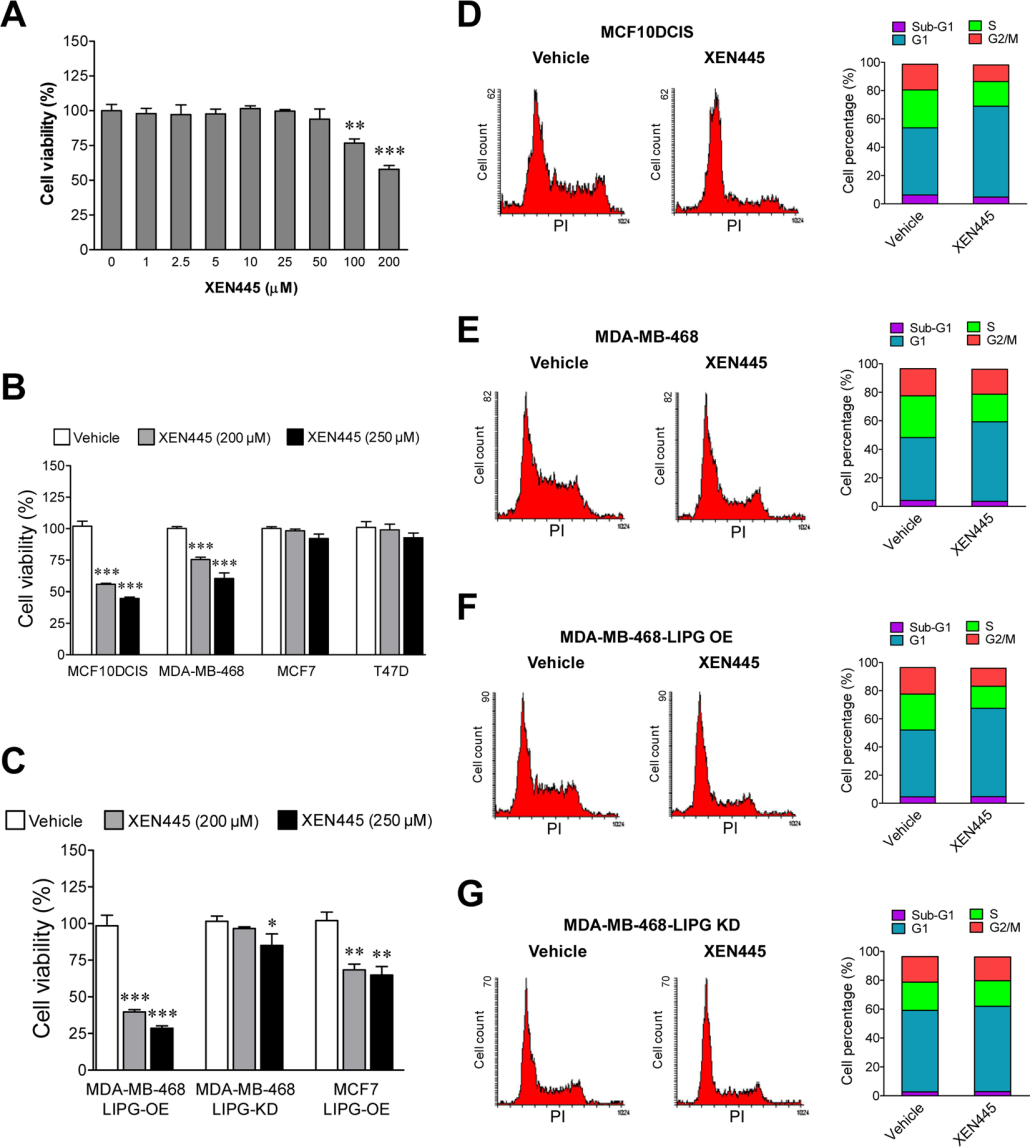
XEN445 selectively inhibits proliferation of LIPG-expressing TNBC cells but not LIPG-negative luminal breast cancer cells. **(A)** XEN445 repressed viability of MCF10DCIS cells in a dose-dependent manner. MCF10DCIS cells were treated with XEN445 at various concentrations as indicated in the bar graph. After 4-day treatment, cells were trypsinized and stained with trypan blue for cell counting. Cell viability was assessed according to viable cell counts. **(B)** Basal-like TNBC cell viability, but not luminal BC cell viability, was inhibited by XEN445 treatment. Two LIPG-expressing basal-like TNBC (MCF10DCIS and MDA-MB-468) and two LIPG-negative luminal BC (MCF7 and T47D) cell lines were treated with either vehicle (DMSO) or XEN445 (200 μM or 250 μM). Cell viability assays were performed on these treated cells after 4-day treatment as described in **(A)**. **(C)** XEN445 suppressed breast cancer cell viability in a LIPG-dependent manner. LIPG-overexpressing and LIPG-knockdown MDA-MB-468 (LIPG-OE and LIPG-KD) and LIPG-overexpressing MCF7 cell lines were treated with either vehicle or XEN445 at 200 μM or 250 μM. Cell viability assays were performed on these treated cells after 4-day treatment as described in **(A)**. Errors shown in **(A–C)** are standard deviation (SD); n = 3; *p < 0.05; **p < 0.01; ***p < 0.001. **(D)** XEN445 induced G1 arrest in MCF10DCIS cells. Cell-cycle profiling analyses of vehicle-treated and XEN445-treated MCF10DCIS cells were performed by staining cells with propidium iodide after four-day treatment. **(E)** XEN445 elicited G1 arrest in MDA-MB-468 cells. Cell-cycle profiling analyses of vehicle-treated and XEN445-treated MDA-MB-468 cells were performed as described in **(D)**. **(F)** Ectopic LIPG overexpression sensitized MDA-MB-468 cells to the inhibitory effect of XEN445 on cell cycle progression. Cell cycle profiling analyses of vehicle-treated and XEN445-treated MDA-MB-468 cells with ectopic LIPG overexpression (LIPG OE) were performed as described in **(D)**. **(G)** LIPG knockdown rendered MDA-MB-468 cells resistant to the inhibitory effect of XEN445 on cell cycle progression. Cell cycle profiling analyses of vehicle-treated and XEN445-treated MDA-MB-468 cells with LIPG knockdown (LIPG KD) were performed as described in **(D)**. For cell cycle profiling bar graphs shown in **(D–F)** and **(G)**, each graph is derived from one representative dataset.

Our previous studies have shown that the enzymatic function of LIPG is required for promoting and sustaining the proliferation of breast cancer cells^16^. Therefore, we expected that XEN445 treatment may inhibit the proliferation of LIPG-expressing breast cancer cells. To test this hypothesis, we performed cell viability assays on MCF10DCIS cells, a LIPG-expressing TNBC cell line^16^, treated with either vehicle (DMSO) or XEN445 (1 to 200 μM). Figure 2A shows that XEN445 (>=100 μM) significantly inhibited the cell viability of MCF10DCIS cells.

To reveal whether XEN445 inhibits cell viability in a LIPG-dependent manner, we treated two LIPG-expressing TNBC cell lines (MCF10DCIS and MDA-MB-468) and two LIPG-deficient luminal breast cancer (LuBC) cell lines (MCF7 and T47D)^16^ with XEN445 at two different doses (200 and 250 μM). The data demonstrate that XEN445 treatment selectively decreased the cell viability of LIPG-expressing TNBC lines rather than LIPG-deficient LuBC lines (Fig. 2B). This result suggests that XEN445-elicited inhibition of cell viability is LIPG-dependent.

To further confirm the result of Fig. 2B, we performed the same XEN445 treatment on LIPG-overexpressing MDA-MB-468 cells, LIPG-knockdown MDA-MB-468 cells and LIPG-overexpressing MCF7 cells. LIPG overexpression and knockdown were confirmed previously^16^. Figure 2C shows that LIPG overexpression sensitized MDA-MB-468 cells to the inhibitory effect of XEN445 when compared to parental cells (Fig. 2B). However, LIPG knockdown rendered MDA-MB-468 resistant to the XEN445 impact (Fig. 2C). Consistently, LIPG overexpression made MCF7 cells sensitive to the inhibitory effect of XEN445 (Fig. 2C), whereas parental MCF7 cells were highly resistant to XEN445 (Fig. 2B). These data, taken together, demonstrate that the antitumor activity of XEN445 is highly LIPG-dependent.

To understand how XEN445 inhibits the viability of TNBC cells, we performed cell cycle analysis of TNBC cells treated with or without XEN445. Figure 2D shows that XEN445 treatment led to an increase in G1 (increased from 47.48% of the control to 64.24%) and decreases in S-G2/M populations (S: decreased from 26.75% of the control to 17.38%; G2/M: decreased from 18.15% of the control to 11.71%) in MCF10DCIS. There was no significant difference between sub-G1 percentages (indicating apoptotic cells) of vehicle-treated (6.27%) and XEN445-treated (4.81%) MCF10DCIS cells, indicating that XEN445 had no significant effect on cell apoptosis. Consistent with MCF10DCIS data, XEN445 treatment of MDA-MB-468 cells induced G1 arrest (G1: increased from 44.14% of the control to 55.74%) and reduced S-G2/M populations (S: decreased from 29.22% of the control to 19.34%; G2/M: decreased from 19.07% of the control to 17.45%), but failed to induce apoptosis (4.16% of the control vs. 3.58%), in MDA-MB-468 cells (Fig. 2E). We further examined whether XEN445 impacts cell cycle with a LIPG-dependent manner. Our FACS’s data demonstrate that ectopic LIPG overexpression sensitized MDA-MB-468 cells to XEN445 treatment, leading to increased G1-phase (increased from 47.55% of the control to 62.80%) and decreased G2/M-phase (decreased from 18.86% of the control to 12.87%) cell populations to a greater extent (Fig. 2F) than those observed in parental cells (Fig. 2E). Similar to parental cells, XEN445 treatment caused a reduction in the S phase by approximately 10% (decreased from 25.52% of the control to 15.71%) (Fig. 2F). In contrast, XEN445-induced differences in G1 (56.38% of control vs. 59.11% of XEN445), S (19.62% of control vs. 17.68% of XEN445) and G2/M (17.69% of control vs. 16.52% of XEN445) phases of MDA-MB-468 cells with LIPG knockdown (Fig. 2G) were minor when compared to parental (Fig. 2E) and LIPG-overexpressing cells (Fig. 2F), indicating that LIPG knockdown rendered MDA-MB-468 cells resistant to the effect of XEN445. These findings, taken together, revealed that XEN445 reduced breast cancer cell viability through G1 cell cycle arrest.

### XEN445 augments the invasiveness of TNBC cells in a LIPG-dependent manner

**Figure 3:**
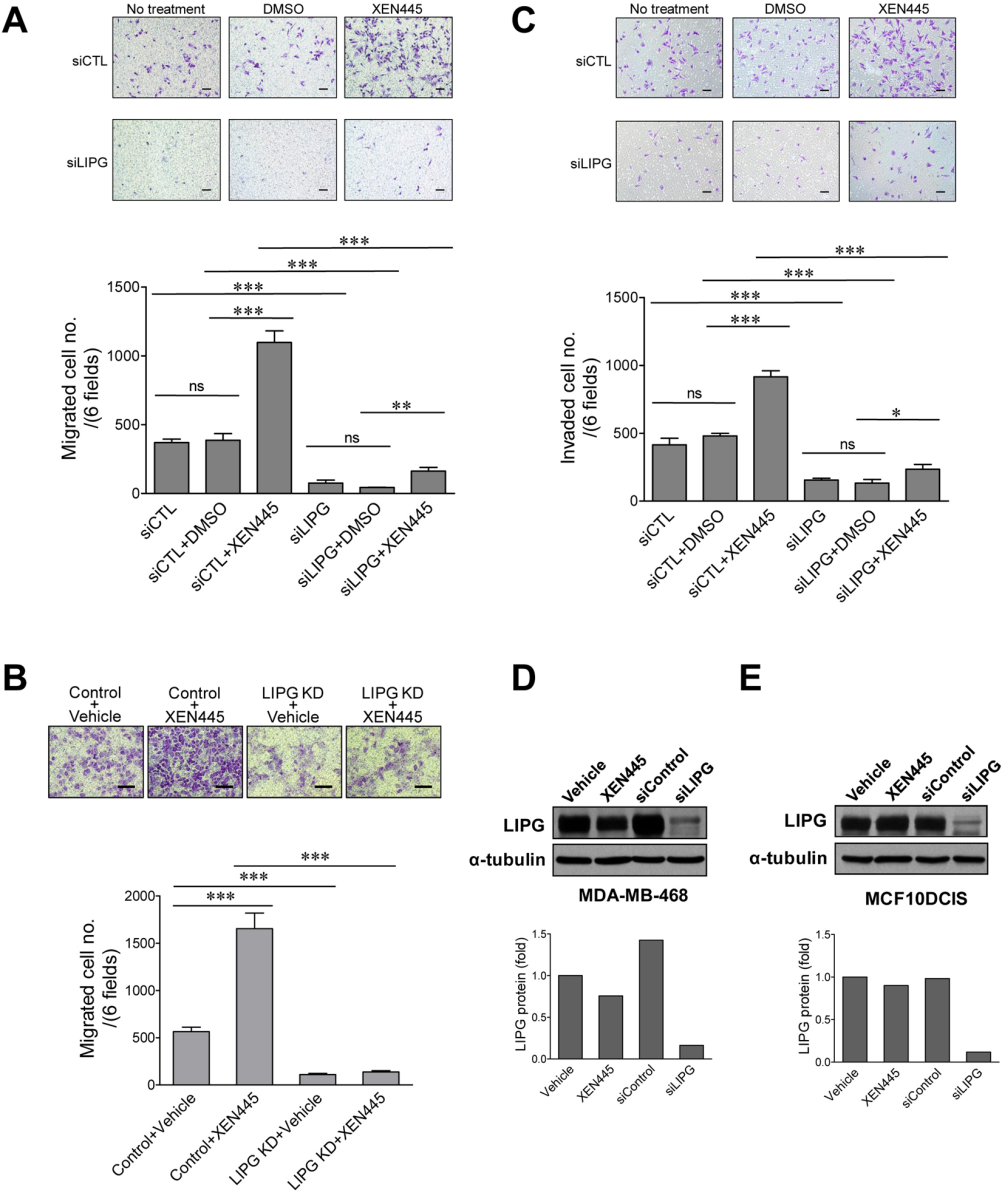
XEN445 promotes invasiveness of TNBC cells in a LIPG-dependent manner. **(A)** XEN445 enhanced migration of MDA-MB-468 cells in a LIPG-dependent manner. MDA-MB-468 cells with or without LIPG knockdown were treated with either vehicle or XEN445 (250 μM) for 3 days and then subjected to migration assays using the transwell method. Control and LIPG siRNA-transfected cells without treatment with either vehicle or XEN445 were included in assays to evaluate the effect of DMSO on cell migration. **(B)** XEN445 promoted migration of MCF10DCIS cells in a LIPG-dependent manner. MCF10DCIS cells with or without LIPG knockdown were treated with either vehicle or XEN445 (250 μM) for 3 days and then subjected to migration assays. **(C)** XEN445 increased invasion of MDA-MB-468 cells in a LIPG-dependent manner. MDA-MB-468 cells were treated as described in **(A)** and then subjected to invasion assays using the matrigel-coated transwell method. Control and LIPG siRNA-transfected cells without treatment with either vehicle or XEN445 were included in assays to evaluate the effect of DMSO on cell invasion. The representative pictures of migrated/invaded cells stained with crystal violet are shown in the top panels. Scale bars indicate 100 μm. Migrated/invaded cells were counted to plot the bar graphs shown in the bottom panels. Errors shown in **(A–C)** are SD; n = 3; *p < 0.05; **p < 0.01; ***p < 0.001. **(D,E)** Western blot analysis of LIPG protein in MDA-MB-468 **(D)** and MCF10DCIS **(E)** cells treated with either vehicle or XEN445, or transfected with either control (siControl) or LIPG siRNA (siLIPG). The respective quantitative western blot data for **(D**,**E)** (n = 1; normalized to α-tubulin) are shown in the bottom panels.

Our previous studies have shown that the invasiveness of TNBC cells is independent of the lipase function of LIPG^16^. To further confirm our findings, we examined the migratory activity of control and LIPG-knockdown MDA-MB-468 cells treated with either vehicle or XEN445 (250 μM) using a Boyden assay. Untreated cells were also included in the study to evaluate the effect of the vehicle (DMSO). Unexpectedly, XEN445 treatment significantly enhanced the migration of MDA-MB-468 cells (~2.84-fold, p < 0.001) when compared to vehicle-treated cells (Fig. 3A). In contrast, LIPG knockdown almost completely abolished this enhancing effect of XEN445 on the migration of MDA-MB-468 (Fig. 3A). The migratory activity of vehicle-treated cells was similar to untreated cells, indicating that DMSO has no significant effect on cell migration. To rule out the possibility that these observed outcomes were cell-line-specific, we performed the same XEN445 treatment experiment on MCF10DCIS cells with or without LIPG knockdown. In line with the result from studying MDA-MB-468 (Fig. 3A), XEN445 treatment also significantly promoted migration of MCF10DCIS cells (~3-fold, p < 0.001) and LIPG knockdown blocked this effect in a same manner (Fig. 3B).

To examine whether XEN445 can also promote invasion of TNBC cells, we performed invasion assays on control and LIPG-knockdown MDA-MB-468 cells treated with either vehicle or XEN445 (250 μM). Consistent with the migration data (Fig. 3A), the invasive activity of MDA-MB-468 cells was also enhanced by XEN445 treatment (~1.9-fold, p < 0.001), which again was abolished by LIPG knockdown (Fig. 3C). The same as the migration data, the vehicle (DMSO) has no significant effect on cell invasion.

To examine whether the XEN445-enhanced TNBC invasiveness resulted from any changes in LIPG protein levels after XEN445 treatment, we performed western blot analysis of LIPG protein expression in MDA-MB-468 and MCF10DCIS cells treated with either vehicle or XEN445 for 4 days. We included control lysates from cells with LIPG knockdown to confirm the specificity of the LIPG antibody. The western blot data demonstrated that XEN445 treatment slightly decreased LIPG protein levels in MDA-MB-468 cells and had no significant impact on LIPG protein levels in MCF10DCIS cells (Fig. 3D,E) (Original western blot images can be found in Supplementary Materials). These results suggest that the XEN445-enhanced TNBC invasiveness does not result from altering LIPG protein levels. In conclusion, we unexpectedly observed that XEN445 enhances the invasiveness of TNBC cells in a LIPG-dependent manner.

### XEN445 has no inhibitory impact on stemness and basal-like features in LIPG-expressing TNBC cells

**Figure 4:**
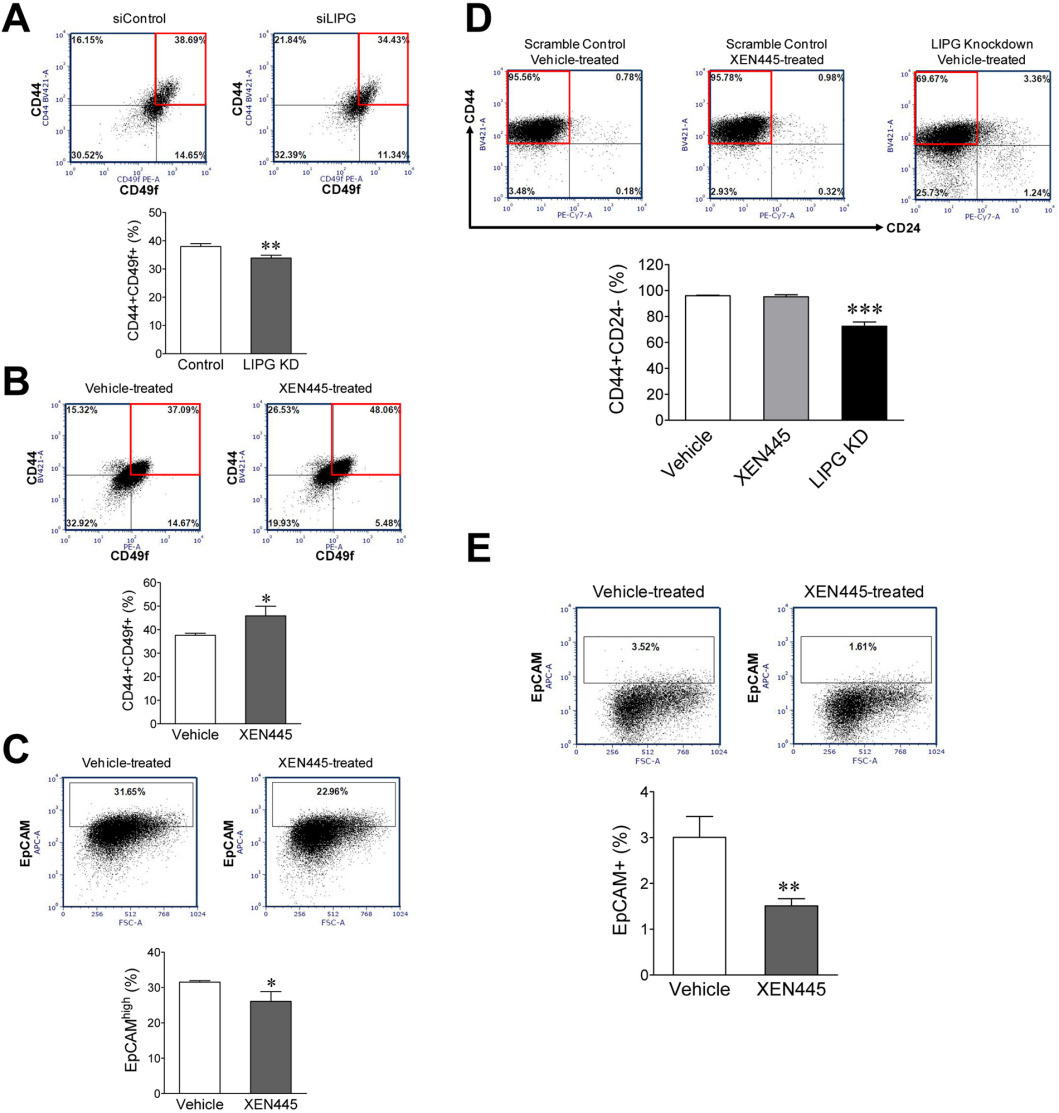
XEN445 has no inhibitory effect on stemness and basal-like features in LIPG-expressing TNBC. **(A)** LIPG knockdown led to a reduction in the CD44+ CD49f+ cell population in MCF10DCIS cells. MCF10DCIS cells were transfected with either control siRNA (siControl) or LIPG siRNA (siLIPG). Three days after siRNA transfection, cells were harvested for flow cytometry analysis of CD44 and CD49f. The boxed area indicates the CD44+ CD49f+ cell population. The CD44+ CD49f+ percentage data were used to plot the bar graph. **(B)** XEN445 treatment led to an increase in the CD44+ CD49f+ cell population in MCF10DCIS cells. MCF10DCIS cells were treated with either vehicle or XEN445 (250 μM) for 4 days and then subjected to flow cytometry analysis of CD44 and CD49f. **(C)** XEN445 enhanced the basal-like feature of MCF10DCIS cells. MCF10DCIS cells were treated with either vehicle or XEN445 as described in **(B)** and then subjected to flow cytometry analysis of EpCAM. The boxed area indicates the EpCAM^high^ cell population. The EpCAM^high^ percentage data were used to plot the bar graph. **(D)** LIPG knockdown, but not XEN445 treatment, led to a reduction in the CD44+ CD24- cell population in MDA-MB-468 cells. Scramble shRNA-expressing (control) and LIPG shRNA-expressing MDA-MB-468 cells were treated with either vehicle or XEN445 (250 μM) as indicated for 4 days and then subjected to flow cytometry analysis of CD44 and CD24. The boxed area indicates the CD44+ CD24- cell population. The CD44+ CD24- percentage data were used to plot the bar graph. **(E)** XEN445 treatment was unable to reduce the basal-like feature of MDA-MB-468 cells. MDA-MB-468 cells treated with either vehicle or XEN445 were subjected to flow cytometry analysis of EpCAM. The boxed area indicates the EpCAM+ cell population. The EpCAM+ cell percentage data were used to plot the bar graph. Errors are SD; n = 3; *p < 0.05; **p < 0.01; ***p < 0.001.

In our previous studies, we demonstrated that the lipase-independent function of LIPG regulates the stemness of TNBC cells^16^. To address the impact of XEN445 on stemness of TNBC cells, we performed flow cytometry analysis of the CD44+ CD49f+ cell population in MCF10DCIS cells, representing a CSC-enriched cell fraction^17^. We first performed the flow cytometry analysis of MCF10DCIS cells with or without LIPG knockdown. LIPG knockdown led to a reduction in the CD44+ CD49f+ cell population from 37.95 ± 1.05% to 33.88 ± 1.00% (p < 0.01) (Fig. 4A). This result indicated that loss of LIPG led to a reduction in the stemness of MCF10DCIS. In contrast, XEN445 treatment (250 μM) significantly increased the percentage of CD44+ CD49f+ cells from 37.61 ± 0.86% to 45.88 ± 4.07% (p < 0.05) (Fig. 4B). To examine the impact of XEN445 on the basal-like characteristics of MCF10DCIS cells (a hallmark of the mesenchymal phenotype in TNBC), we performed flow cytometry analysis of EpCAM (a well-known epithelial/luminal protein marker inversely related to the cell basal-like feature) on MCF10DCIS cells treated with either vehicle or XEN445. As shown in Fig. 4C, XEN445 treatment reduced the percentage of EpCAM^high^ cells in MCF10DCIS from 31.51 ± 0.40% to 26.09 ± 2.73% (p < 0.05), indicating that XEN445 promoted the basal-like phenotype of MCF10DCIS cells. We were surprised for this data because our previous LIPG knockdown experiments showed that LIPG knockdown significantly altered the basal-like characteristic of MCF10DCIS cells to the luminal phenotype^16^.

To confirm whether the findings described above can be recapitulated in MDA-MB-468 cells, we first performed flow cytometry analysis of the CD44+ CD24- cell population, representing a CSC-enriched cell fraction in MDA-MB-468. We found that XEN445 treatment had no impact on the CD44+ CD24- cell population in MDA-MB-468 (96.00 ± 0.41% of control vs. 95.27 ± 1.51% of XEN445), whereas LIPG knockdown significantly decreased the percentage of CD44+ CD24- cells (from 96.00 ± 0.41% of the control to 72.54 ± 3.24%, p < 0.001) (Fig. 4D). Moreover, flow cytometry analysis of EpCAM-positive (EpCAM+) cells showed that XEN445 was unable to change the basal-like feature of MDA-MB-468 cells to the luminal phenotype because we did not observe an increase in the EpCAM+ cell percentage (Fig. 4E). Actually, EpCAM analysis showed a reverse result that XEN445 treatment gave rise to a reduction in the EpCAM+ cell population (decreased from 3.01 ± 0.45% of the control to 1.51 ± 0.16%, p < 0.01), similar to the result from the MCF10DCIS study (Fig. 4C). These findings indicate that XEN445 has no inhibitory effect on stemness and basal-like traits in LIPG-expressing TNBC cells.

### XEN445 suppresses CSC self-renewal of LIPG-expressing TNBC

**Figure 5:**
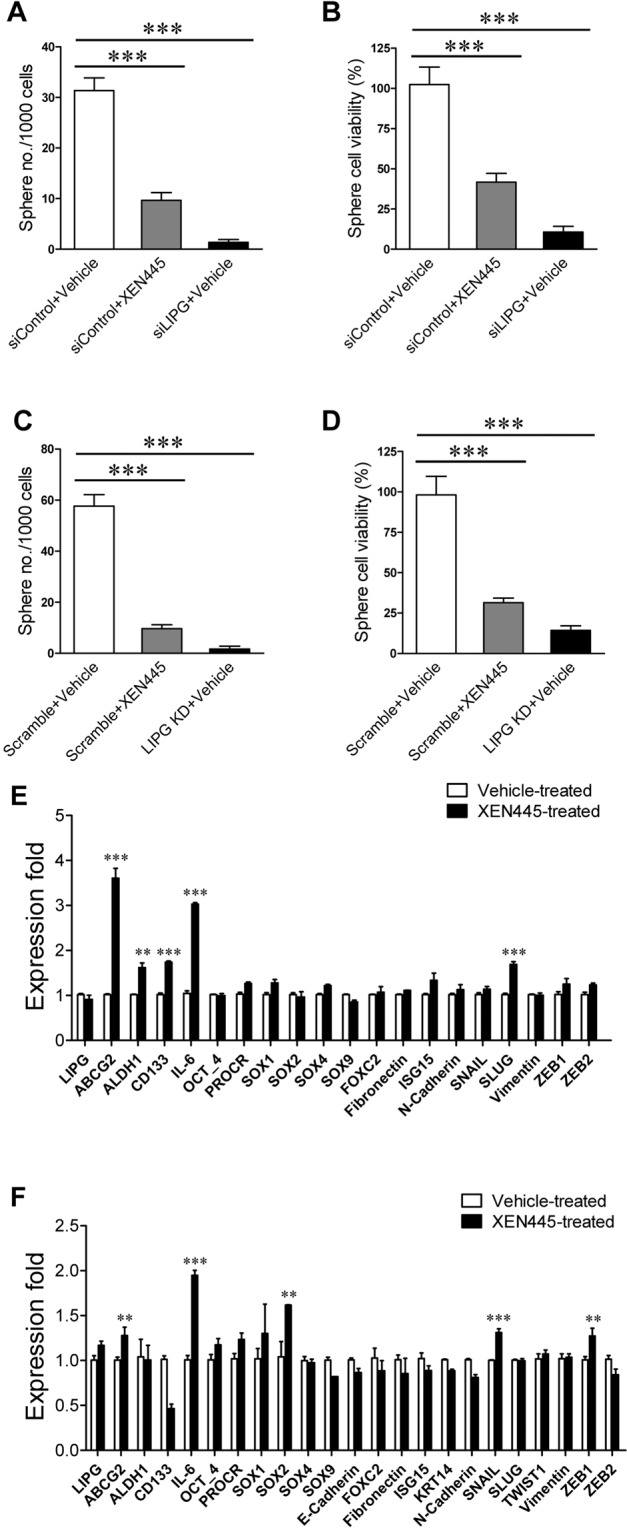
XEN445 impedes CSC self-renewal of LIPG-expressing TNBC cells but has no inhibitory effect on expression of stemness and EMT programming genes. **(A,B)** XEN445 attenuated self-renewal of cancer stem cells in MCF10DCIS. MCF10DCIS cells were transfected with either control siRNA (siControl) or LIPG siRNA (siLIPG). 24 hours after transfection, single cells were prepared and then replated in the ultra-low attachment well containing the serum-free sphere culture medium with either vehicle or XEN445 as indicated for sphere formation. After a week, the numbers of formed tumorspheres were counted according to the sphere size criterion described in “Materials and Methods”. After sphere counting, tumorspheres were dissociated into single cells using accutase for cell counting. The obtained tumorsphere and single cell number data were used to plot the bar graphs of sphere formation (shown in **A**) and cell viability (shown in **B**). **(C,D)** XEN445 inhibited self-renewal of cancer stem cells in MDA-MB-468. Scramble shRNA-expressing (control) and LIPG shRNA-expressing MDA-MB-468 cells were harvested and then replated in the ultra-low attachment well containing the serum-free sphere culture medium with either vehicle or XEN445 as indicated for sphere formation. After a week, the numbers of formed tumorspheres were counted as described above. After sphere counting, tumorspheres were dissociated into single cells as described above. The obtained tumorsphere and single cell number data were used to plot the bar graphs of sphere formation (shown in **C**) and cell viability (shown in **D**). **(E)** Expression profiling analysis of stemness-related and EMT programming genes in vehicle-treated and XEN445-treated MDA-MB-468 cells. **(F)** Expression profiling analysis of stemness-related and EMT programming genes in vehicle-treated and XEN445-treated MCF10DCIS cells. Errors are SD; n = 3; **p < 0.01; ***p < 0.001.

Self-renewal of CSCs is a process of renewing CSC, which involves both activated stemness and cell division. The sphere formation assay is a frequently performed method to measure the self-renewal capacity of CSCs. To examine the impact of XEN445 on self-renewal of CSCs in LIPG-expressing TNBC, we performed sphere formation assays on MCF10DCIS cells with either XEN445 treatment or LIPG knockdown compared to control cells. We found that the total number of formed CSC spheres from MCF10DCIS cells was significantly decreased by XEN445 treatment (~ 69% reduction, p < 0.001) (Fig. 5A). Consistently, the total number of sphere cells was decreased by XEN445 treatment (~59% reduction, p < 0.001) (Fig. 5B). Furthermore, LIPG knockdown in MCF10DCIS cells dramatically reduced the total numbers of CSC spheres (~ 96% reduction, p < 0.001)(Fig. 5A) and sphere cell viability (~90% reduction, p < 0.001) (Fig. 5B). These findings demonstrate that XEN445 attenuates the self-renewal of CSCs in MCF10DCIS.

To determine the effect of XEN445 on CSC self-renewal of MDA-MB-468 cells, we performed sphere formation assays on XEN445-treated cells compared to control and LIPG-knockdown cells. Consistent with the result from the MCF10DCIS study (Fig. 5A), the treatment of MDA-MB-468 with XEN445 significantly decreased the total number of formed CSC spheres (~83% reduction, p < 0.001) (Fig. 5C). In line with the sphere data, the total number of viable MDA-MB-468 sphere cells was significantly decreased by XEN445 treatment (~68% reduction, p < 0.001) (Fig. 5D). Similarly, LIPG knockdown in MDA-MB-468 cells led to dramatic decreases in the total numbers of formed CSC spheres (~97% reduction, p < 0.001) and viable sphere cells (~85% reduction, p < 0.001) (Fig. 5C,D). Given that the flow cytometry data showed that XEN445 had no inhibitory impact on the subpopulation of TNBC CSCs (Fig. 4), the data from sphere formation assays suggest that XEN445 inhibited self-renewal of CSCs in LIPG-expressing TNBC through suppressing the cell division of CSCs, but not CSC stemness properties.

### XEN445 promotes the expression of stemness-related and EMT-programming genes in LIPG-expressing TNBC cells

Our previous studies showed that LIPG knockdown inactivated both enzymatic and non-enzymatic functions of LIPG and downregulated the expression of genes involved in stemness and EMT programming^16^. Therefore, we next examined the impact of pharmacological inhibition of LIPG phospholipase activity by XEN445 on the expression of LIPG and these stemness-related and EMT-related genes. We first performed qRT-PCR analysis of expression of ten stemness-related and twelve EMT-programming-related genes in vehicle-treated and XEN445-treated MDA-MB-468 cells. As shown in Fig. 5E, XEN445 treatment led to the upregulated expression of five genes (at least>1.5-fold, p < 0.001) among 20 genes with detectable expression. These 5 genes include four stemness-related genes (ABCG2, >3-fold; ALDH1, >1.5-fold; CD133, >1.5-fold; IL-6, > 3-fold) and one EMT-related gene (SLUG, >1.5-fold) (Fig. 5E). We unexpectedly observed that XEN445 treatment selectively upregulated the expression of some stemness- and EMT-related genes. We further performed qRT-PCR to verify our results in MCF10DCIS cells treated with either vehicle or XEN445. We observed a similar result that XEN445 treatment led to upregulated expression of three stemness-related (ABCG2, ~1.3-fold, p < 0.01; IL-6, ~2.0-fold, p < 0.001; SOX2, ~1.6-fold, p < 0.01) and two EMT-related (SNAIL, ~1.3-fold, p < 0.001; ZEB1, ~1.3-fold, p < 0.01) genes albeit to a lesser degree (Fig. 5F). The only exception is CD133, a stemness-related gene, which showed downregulated expression following XEN445 treatment (Fig. 5F). These results demonstrate that XEN445 mainly upregulates stemness-related genes.

### XEN445 impedes *in vivo* tumor growth of basal-like TNBC cells

**Figure 6:**
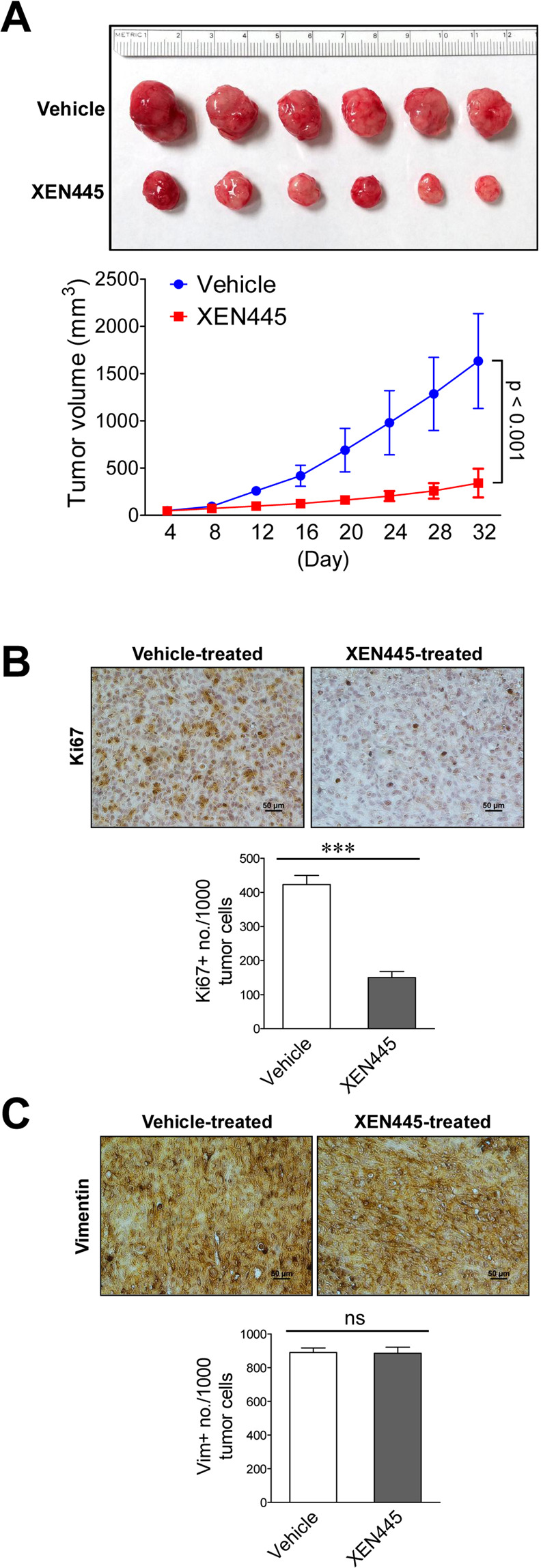
XEN445 therapy retards *in vivo* tumor growth of MDA-MB-468 cells but has no inhibitory impact on the basal-like phenotype of formed tumors. **(A)** The xenograft tumor growth of MDA-MB-468 cells in nude mice was suppressed by XEN445 therapy. Fourth mammary fat pads of 2-month-old female nude mice were transplanted with MDA-MB-468 cells. After cell transplantation, mice were treated with either vehicle or XEN445 (50 mg/kg) for 32 days. The picture of harvested tumors is shown in the top panel and the plotted tumor growth curves are shown in the bottom panel. **(B)** Immunohistochemistry analysis of Ki67 in MDA-MB-468 xenograft tumors harvested from mice treated with either vehicle or XEN445. Representative staining pictures are shown. Ten randomly selected fields for each stained tissue section were used to count Ki67-positive and total tumor cells. Ki67 positivity was expressed as the Ki67-positive cell number per 1000 counted tumor cells. The quantitative bar graph was plotted based on the counting results from three different stained tumor tissue sections prepared from three transplanted nude mice for each xenograft group. Errors are SD; n = 3; ***p < 0.001. **(C)** Immunohistochemistry analysis of vimentin in MDA-MB-468 xenograft tumors harvested from mice treated with either vehicle or XEN445. Representative staining pictures are shown. Scale bars indicate 50 μm. The quantitative bar graph for the vimentin staining data was generated as described in **(B)**. ns: not significant.

To address the impact of pharmacological inhibition of LIPG enzymatic function by XEN445 on TNBC tumor growth *in vivo*, we performed XEN445 therapy (50 mg/kg) on nude mice with MDA-MB-468 xenograft tumors. Consistent with the result from *in vitro* cell studies shown in Fig. 2B, XEN445 treatment significantly inhibited tumor growth in nude mice (p < 0.001) (Fig. 6A). To determine whether tumor cell proliferation is inhibited *in vivo* by XEN445 treatment, we performed immunohistochemistry (IHC) analysis of Ki67, a cell proliferation marker, on vehicle- and XEN445-treated tumors. In line with the tumor growth data, XEN445 therapy significantly decreased the number of Ki67-positive cells in xenograft tumors (150 ± 18/1000 tumor cells, n = 3, p < 0.001) when compared to vehicle-treated tumors (423 ± 27/1000 tumor cells, n = 3) (Fig. 6B). To examine the EMT status of XEN445-treated xenograft tumors, we performed IHC analysis of vimentin on isolated tumors treated with either vehicle or XEN445. As shown in Fig. 6C, there was no significant difference in vimentin staining between vehicle- and XEN445-treated tumors. This finding suggests that XEN445 therapy has no inhibitory impact on the EMT/mesenchymal phenotype of MDA-MB-468 xenograft tumors, consistent with the result from the qRT-PCR study (Fig. 5E). These *in vitro* and *in vivo* findings from XEN445 therapy studies contrast with our previous findings from LIPG knockdown studies showing that LIPG loss-of-function led to downregulation of vimentin expression in MDA-MB-468 cells^16^.

## Discussion

Several studies have revealed that histone H3 K36 demethylase KDM4A, caspase-8, and lysyl oxidase have enzymatic and non-enzymatic functions^18–20^. Mechanistically, these enzymes execute their non-enzymatic functions through protein-protein interactions. Our previous studies have shown that LIPG also possesses both enzymatic and non-enzymatic functions in breast cancer cells^16^. The phospholipase function of LIPG is responsible for supporting cell growth and promoting cell proliferation rate. In contrast, the phospholipase-independent function of LIPG is involved in oncogenic DTX3L-ISG15 signaling and promotes invasiveness, stemness and basal/EMT features of breast cancer cells^16^. Although the mechanism by which LIPG executes its non-enzymatic function is unknown, it is likely through protein-protein interactions.

The only currently approved targeted therapy for TNBC is the immunotherapy with atezolizumab for patients whose tumors express PD-L1, which was found to increase progression-free survival. Since our prior studies have shown that LIPG is essential for the malignancy and metastasis of TNBC^16^, it is clinically imperative to investigate the therapeutic effects of currently available chemical inhibitors targeting LIPG. In this study, we for the first time explored the therapeutic impacts of XEN445, a chemical inhibitor specific to the phospholipase activity of LIPG^8^, on TNBC malignancy. We first examined the effect and IC50 of XEN445 on LIPG phospholipase activity. Consistent with the previous finding^8^, XEN445 specifically inhibited the enzymatic activity of LIPG in our cell-based LIPG enzymatic activity assay system, and IC50 is approximate 2 μM, which is approximately 9-fold higher than the previously reported IC50^8^. Furthermore, we found that the high dose of XEN445 (200 μM) was needed to observe its significantly inhibitory effect in cell function studies. This dose is approximately 100-fold higher than IC50 observed in the LIPG enzymatic study. The serum in the cell culture medium is a possible cause leading to the need of the high dose of XEN445 in cell function studies as serum proteins are reported to bind XEN445^8^ and therefore it is expected that serum affects the concentration of the free form of XEN445 in the cell culture medium.

With regard to the impact of XEN445 on malignancy of breast cancer cells, we found that in concordance with findings from studies of the catalytically inactive LIPG mutant^16^, XEN445 suppressed TNBC cell proliferation and CSC self-renewal, but not other TNBC properties such as invasiveness, and EMT/basal-like features. In line with these *in vitro* findings, XEN445 therapy inhibited *in vivo* tumor growth of TNBC xenografts, but not their basal-like characteristics. Therefore, our studies suggest that pharmacologically inhibiting LIPG phospholipase activity is not sufficient to hinder every aspect of TNBC malignancy.

In addition to our finding that XEN445 was unable to impede the non-enzymatic function of LIPG, our studies have revealed another important finding that XEN445 actually enhanced the non-enzymatic function of LIPG, evidenced by XEN445-induced invasiveness, stemness and EMT/basal-like features. It remains unknown how XEN445 can enhance tumor cell invasiveness. However, our previous studies have shown that ectopic overexpression of the catalytically inactive LIPG mutant also enhanced migration and stemness of luminal breast cancer cells to a greater degree than overexpression of wild-type LIPG^16^. These prior findings indicate that inactivating LIPG phospholipase activity promotes the non-enzymatic function of LIPG. Based on our studies, we hypothesize that LIPG phospholipase activity has an intrinsically inhibitory effect on the non-enzymatic function of LIPG in a direct and/or indirect manner. If this is true, inactivating both enzymatic and non-enzymatic functions of LIPG will be essential for inhibiting the whole oncogenic function of LIPG in breast cancer.

Accumulating evidence indicates that disseminated tumor cells (DTCs) can acquire stem-cell-like properties as well as the mesenchymal/EMT feature and their quiescent stemness (also known as stem-cell dormancy) is activated during their metastasis and after DTCs settle down at distant tissues^21^. These stem-cell-like DTCs can stay in the dormant state at distant tissues for many years before they are activated to proliferate and differentiate in the formation of metastatic tumors^21^. The essential role of LIPG in TNBC metastasis ^16^suggests that both enzymatic and non-enzymatic functions of LIPG participate in modulating the metastatic process, the dormancy of DTCs and metastatic outgrowth in a highly plastically regulatory manner. Our study suggests that inhibition of LIPG phospholipase activity only impedes primary tumor growth as well as metastatic outgrowth and has no impact on dormant DTCs and quiescent primary tumor cells. Furthermore, blocking LIPG enzymatic activity may potentially promote CSC properties as well as EMT/basal-like features in tumor cells and induce the dormancy of DTCs. Therefore, the development of new therapeutic agents that can target both enzymatic and non-enzymatic functions of LIPG is necessary in the future for TNBC therapy.

## Materials and Methods

### Cell culture and XEN445

We obtained breast cancer cell lines, including MDA-MB-468, T47D and MCF7 from ATCC (American Type Culture Collection, Manassas, VA). The human DCIS cell line MCF10DCIS.COM (MCF10DCIS) was purchased from Asterand USA (Detroit, MI). These cell lines were cultured according to manufacturer’s instructions. We obtained XEN445 from Xenon Pharmaceuticals INC (Burnaby, Canada).

### LIPG enzymatic assay

Both parental and LIPG-overexpressing MDA-MB-468 cell lines were analyzed by the LIPG enzymatic assay. To perform this assay, single cells were prepared and washed with the assay buffer (HBSS with 25 mM HEPES), and cells were eventually suspended in an appropriate volume of the assay buffer. Cells were diluted with the assay buffer to make the 2X cell prep (6 × 10^5^/mL). To make the 2X working substrate solution, 1 mM PED-A1 (Thermo Fisher Scientific), a specific substrate for phospholipase A1,was diluted with HBSS-HEPES (1:125 dilution) to 8 μM. For analysis of the inhibitory effect of XEN445, a series of 2 times XEN445 doses in the 2X working substrate solution were prepared. 50 μl of the 2X working substrate solution with or without XEN445 was loaded into each well of the 96-well plate. 50 μl of the cell suspension (3 × 10^4^) from the 2X cell prep was loaded into each well and mixed with the working substrate solution. Enzymatic assays were performed at 37 °C in a kinetic mode (488 nm for excitation, 520 nm for emission) using the setting of 30 cycles (1 cycle/min). For the calculation of LIPG enzymatic activity, the measured fluorescence data from 468-LIPG OE cell samples were subtracted by the average basal fluorescence data from DMSO-treated 468-CTRL cell samples to obtain LIPG OE-contributed fluorescence measurements, and then the average fluorescence value (after subtraction) from DMSO-treated 468-LIPG OE cell samples was set as default 100% to calculate the relative LIPG enzymatic activities of other treated 468-LIPG OE cell samples.

### siRNA transfection

The siRNA transfection was performed with 40 nM of siRNA using Lipofectamine RNAiMAX (Thermo Fisher Scientific) according to manufacturer’s instructions. siRNAs were obtained from Sigma-Aldrich. The siRNA sequences used in the study are: siControl, 5′-UAACUCGCUCGAAGGAAUC-3′; siLIPG, 5′-GCCGCAAGAACCGUUGUAA-3′^16^.

### Establishment of stable LIPG-overexpression and LIPG-knockdowncell lines

Stable LIPG-overexpression and LIPG-knockdown MDA-MB-468 cell lines were established as described previously^16^.

### Cell viability assay

2 × 10^4^ cells were seeded into the well of a 6-well cell culture plate with culture media and cultured overnight. Cells were then treated with either vehicle (DMSO) or XEN445 for four days. After four-day treatment, cells were trypsinized and spun down. The cells were resuspended in the 0.5 ml medium and 10 μl of cells was taken for cell counting by staining with trypan blue. The viability of drug-treated cells relative to vehicle-treated cells was determined by the ratio of drug- and vehicle-treated cell counts.

### Cell cycle analysis

Cell cycle analysis was performed as previously described^22^. In brief, trypsinized cells at a density of 10^6^ cells/ml in phosphate buffered saline (PBS) were fixed by 70% ethanol. Ethanol-fixed cells were stained in the staining buffer made of propidium iodide (0.4 mg/ml), RNase A (0.1 mg/ml) and 0.1% Triton X-100 in PBS. The cell-cycle profile and sub-G1 fraction were analyzed by flow cytometry using the Becton Dickinson FACSAria II system (Franklin Lakes, NJ).

### Migration and invasion assays

Transwell-based migration and invasion assays were implemented as previously described^23^.

### Western blot analysis

Western blot analysis was performed as previously described^16^. Protein expression of LIPG was examined by western blotting using a mouse antibody against LIPG (Abcam, ab56493). Protein expression was detected by chemiluminescence (ECL, Pierce). Expression of α-tubulin (Thermo Fisher Scientific) was used as a protein loading control. Western blot data were quantified by densitometric analysis of autoradiograms, using a computerized densitometer (Typhoon System; Molecular Dynamics, Inc., Sunnyvale, CA). The quantitative protein level data were normalized by the α-tubulin protein levels.

### Flow cytometry analysis

Cells were stained with the following antibodies from BioLegend (San Diego, CA): brilliant violet (BV) 421-conjugated anti-CD44, phycoerythrin (PE)-conjugated anti-CD24, phycoerythrin (PE)-conjugated anti-CD49f and allophycocyanin (APC)-conjugated anti-EpCAM. Flow cytometry analysis of stained cells was performed using FACSAria II (Becton Dickinson, Franklin Lakes, NJ) as previously described^23^.

### Sphere formation assay

We performed sphere formation assays as previously described^23^. After one-week sphere culture, formed primary spheres were counted under a microscope according to the sphere size criterion (≥100 μm). For sphere cell viability assays, spheres were collected and dissociated with accutase (BioLegend) for obtaining single sphere cells. Collected sphere cells were spun down and resuspended in the 0.2 ml sphere culture medium. 10 μl of dissociated, single sphere cells was taken for cell counting by staining with trypan blue. The viability of treated sphere cells relative to control sphere cells was determined by the ratio of treated and control sphere cell counts.

### RNA isolation and quantitative RT-PCR (qRT-PCR) analysis

Total RNA of cultured cells was isolated using the Ambion TRIzol reagent (Thermo Fisher Scientific, Halethorpe, MD) according to manufactures’ instructions. qRT-PCR analysis of mRNA expression was performed as described previously with normalization to GAPDH^23^. The sequence information of gene primers used in qRT-PCR experiments has been included in our publications^16,17^.

### *In vivo* tumorigenicity assay

The fourth mammary glands of 8-week-old female nude mice were transplanted with MDA-MB-468 cells (1 × 10^6^ cells per injection) for xenograft mammary tumor formation. After cell transplantation, mice were subsequently split into two groups that were treated with vehicle and XEN445 (50 mg/kg), respectively, via intraperitoneal (IP) injection on the same day and later three times per week. Athymic nude mice (NU/NU mice, Stock No: 002019) were obtained from the Jackson Laboratory (Bar Harbor, ME). The length and width of tumors were measured every four days with a caliper to calculate tumor volume using the formula: V = 1/2 (Length × Width^2^)^24^. At the endpoint, xenograft tumors were isolated and processed for immunohistochemistry analysis. Xenograft tumor experiments were performed according to the animal protocol approved by the Institutional Animal Care and Use Committee (IACUC) of the University of Maryland School of Medicine, which is in accordance with the guidelines established by the USPHS.

### Immunohistochemistry assay

The immunohistochemistry (IHC) analysis was performed using the avidin biotin peroxidase complex (ABC) method as previously described^17^. Anti-Ki67 (27309-1-AP) and anti-vimentin (10366-1-AP) rabbit polyclonal antibodies used in IHC experiments were obtained from Proteintech (Rosemont, IL).

### Statistical analysis

Statistical analysis was performed as previously described^16^. Data are presented as mean ± S.D. Statistical analysis of general experimental datasets was performed by Student’s t test. Statistical analysis of tumor growth curves was performed by Two-way ANOVA. The *p* values of <0.05 were considered significant. Data were analyzed using the GraphPad Prism software (version 6.0; GraphPad Software, Inc, La Jolla, CA).

### DVA/US Government disclaimer

The contents do not represent the views of the U.S. Department of Veterans Affairs or the United States Government.

## Supplementary information

Supplemantary information.

## References

[1] Jaye M (1999). A novel endothelial-derived lipase that modulates HDL metabolism. Nat. Genet..

[2] Hirata K (1999). Cloning of a unique lipase from endothelial cells extends the lipase gene family. J. Biol. Chem..

[3] Ishida T (2003). Endothelial lipase is a major determinant of HDL level. J. Clin. Invest..

[4] Maugeais C (2003). Dose-dependent acceleration of high-density lipoprotein catabolism by endothelial lipase. Circulation.

[5] Goodman KB (2009). Discovery of potent, selective sulfonylfuran urea endothelial lipase inhibitors. Bioorg. Med. Chem. Lett..

[6] O’Connell DP (2012). Design and synthesis of boronic acid inhibitors of endothelial lipase. Bioorg. Med. Chem. Lett..

[7] Greco MN (2013). A thiocarbamate inhibitor of endothelial lipase raises HDL cholesterol levels in mice. Bioorg. Med. Chem. Lett..

[8] Sun S (2013). Discovery of XEN445: a potent and selective endothelial lipase inhibitor raises plasma HDL-cholesterol concentration in mice. Bioorg. Med. Chem..

[9] Perou CM (2000). Molecular portraits of human breast tumours. Nature.

[10] Sorlie T (2001). Gene expression patterns of breast carcinomas distinguish tumor subclasses with clinical implications. Proc Natl Acad Sci USA.

[11] Rakha EA (2009). Triple-negative breast cancer: distinguishing between basal and nonbasal subtypes. Clin Cancer Res.

[12] Sorlie T (2003). Repeated observation of breast tumor subtypes in independent gene expression data sets. Proc Natl Acad Sci USA.

[13] Badve S (2011). Basal-like and triple-negative breast cancers: a critical review with an emphasis on the implications for pathologists and oncologists. Mod Pathol.

[14] Lehmann BD (2011). Identification of human triple-negative breast cancer subtypes and preclinical models for selection of targeted therapies. J Clin Invest.

[15] Honeth, G. *et al*. The CD44+/CD24- phenotype is enriched in basal-like breast tumors. *Breast Cancer Res* **10**, R53 (2008). 10.1186/bcr2108 PMC2481503 18559090

[16] Lo PK (2018). LIPG signaling promotes tumor initiation and metastasis of human basal-like triple-negative breast cancer. Elife.

[17] Lo PK (2017). Tumor-associated myoepithelial cells promote the invasive progression of ductal carcinoma *in situ* through activation of TGFβ signaling. J. Biol. Chem..

[18] Trackman PC (2016). Enzymatic and non-enzymatic functions of the lysyl oxidase family in bone. Matrix Biol.

[19] Colmenares SU (2017). Drosophila Histone Demethylase KDM4A Has Enzymatic and Non-enzymatic Roles in Controlling Heterochromatin Integrity. Dev Cell.

[20] Henry CM, Martin SJ (2017). Caspase-8 Acts in a Non-enzymatic Role as a Scaffold for Assembly of a Pro-inflammatory “FADDosome” Complex upon TRAIL Stimulation. Mol Cell.

[21] Weidenfeld K, Barkan D (2018). EMT and Stemness in Tumor Dormancy and Outgrowth: Are They Intertwined Processes?. Front. Oncol..

[22] Lo PK, Huang SZ, Chen HC, Wang FF (2004). The prosurvival activity of p53 protects cells from UV-induced apoptosis by inhibiting c-Jun NH2-terminal kinase activity and mitochondrial death signaling. Cancer Res.

[23] Lo PK (2016). Dysregulation of the BRCA1/long non-coding RNA NEAT1 signaling axis contributes to breast tumorigenesis. Oncotarget.

[24] Jensen MM, Jørgensen JT, Binderup T, Kjaer A (2008). Tumor volume in subcutaneous mouse xenografts measured by microCT is more accurate and reproducible than determined by 18F-FDG-microPET or external caliper. BMC Med. Imaging.

