# Supplementary material for: Inhibition of LIPG phospholipase activity suppresses tumor formation of human basal-like triple-negative breast cancer
Source: Sci Rep. 2020 Jun 2;10:8911. doi: 10.1038/s41598-020-65400-7 (PMC7265491; doi:10.1038/s41598-020-65400-7)

Inhibition of LIPG phospholipase activity suppresses tumor formation of human basal-like triple-negative breast cancer

Pang-Kuo Lo, Yuan Yao & Qun Zhou

Original WB images for Figure 3D and 3E

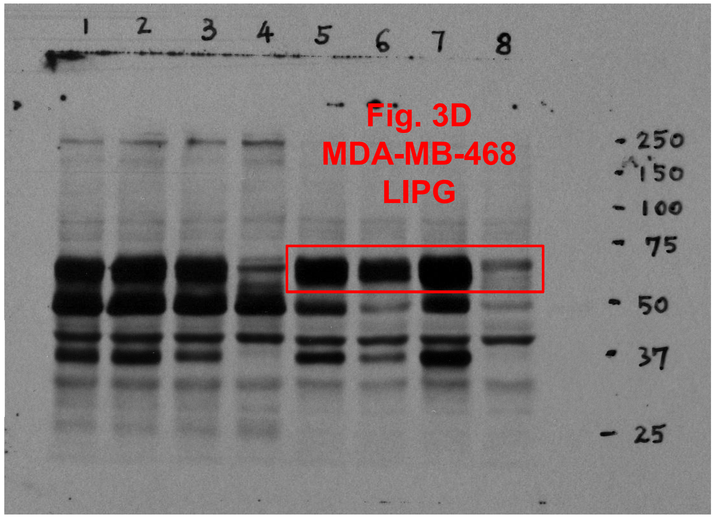

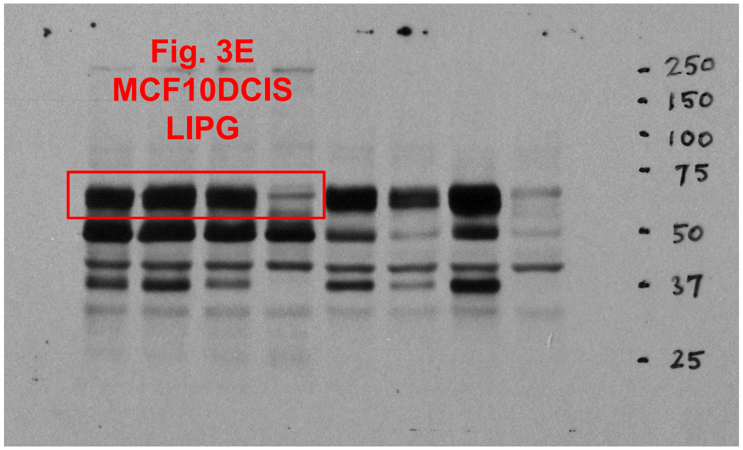

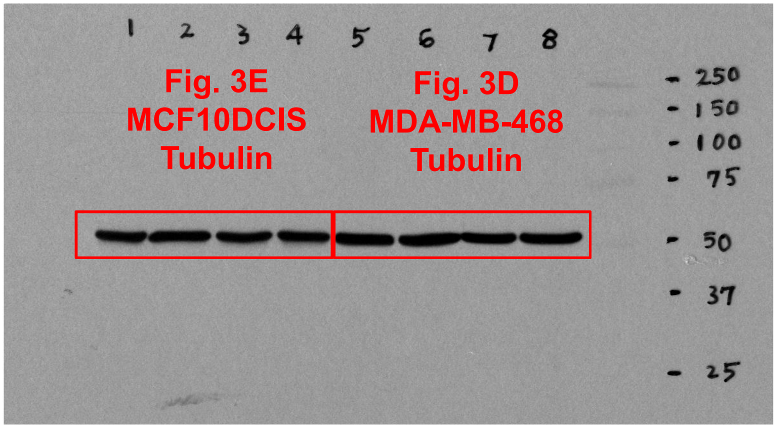

Supplement: Supplementary file 1 — Supplemantary information. [file 41598_2020_65400_MOESM1_ESM.pdf]
